# Preserved Subcutaneous Implantable Cardioverter-defibrillator Function Following Septal Myectomy and Coronary Artery Unroofing in a Pediatric Patient with Severe Hypertrophic Cardiomyopathy

**DOI:** 10.19102/icrm.2025.16074

**Published:** 2025-07-15

**Authors:** Leon Przybylowski, John J. Parent, Jeremy L. Herrmann, Adam C. Kean

**Affiliations:** 1 Department of Pediatrics, Division of Pediatric Cardiology, The Ohio State University School of Medicine, Columbus, OH, USA; 2 Department of Pediatrics, Division of Pediatric Cardiology, Indiana University School of Medicine, Indianapolis, IN, USA; 3 Department of Surgery, Section of Cardiothoracic Surgery, Indiana University School of Medicine, Indianapolis, IN, USA

**Keywords:** Hypertrophic cardiomyopathy, pediatrics, subcutaneous implantable cardioverter-defibrillator

## Abstract

Hypertrophic cardiomyopathy (HCM) is an inherited disease present in 1 in 500 individuals and is the most common cause of sudden cardiac death in children. We present the case of a 17-year-old boy with HCM and a primary prevention subcutaneous implantable cardioverter-defibrillator (S-ICD) who developed left ventricular outflow tract obstruction and a myocardial bridge of the left anterior descending coronary artery. The patient underwent a septal myectomy/myotomy and muscular bridge unroofing. The S-ICD system was undisturbed during the surgery, with no loss of function. Septal myectomy may be accomplished in pediatric HCM patients following optimal S-ICD placement with maintained S-ICD function.

## Introduction

Hypertrophic cardiomyopathy (HCM) is an inherited cardiac disease present in at least 1 in 500 individuals.^1^ HCM is the most common cause of sudden cardiac death (SCD) in children and young athletes, and, in those with HCM, the incidence of SCD has been estimated to range between 1% and 7%, leading to established guidelines for the placement of primary and secondary prevention implantable cardioverter-defibrillators (ICDs).^1–3^ Transvenous ICDs and subcutaneous ICDs (S-ICDs) are the most common systems placed, and recent studies demonstrate that the two are comparable in treatment efficacy and complications.^4–6^ In addition to the risk of SCD, symptomatic left ventricular outflow tract (LVOT) obstruction causes additional morbidity. A subset of patients with LVOT obstruction may require myectomy.^1^ There have been reports of preservation of S-ICDs following a sternotomy, including a case series with historical controls.^7–9^ There is, however, limited literature with mixed results of patients retaining their S-ICDs following a myectomy and no reports specifically addressing a coronary artery myocardial bridge.^10,11^ We report an adolescent patient who had his S-ICD preserved despite undergoing a myectomy and an unroofing of a myocardial bridge of his left anterior descending coronary artery (LAD) almost 5 years after S-ICD implantation.

The Indiana University Human Research Protection Program (IU HRPP) determined that the study is not human subjects research and does not require further review (protocol #25956; January 17, 2025). We made every reasonable effort to follow-up with the patient both for clinical care and consent to publish de-identified case information. Further follow-up was not successful.

## Case presentation

A 17-year-old boy was initially diagnosed with HCM at 10 years of age. His father was subsequently diagnosed with HCM, and there was no family history of sudden cardiac arrest. The patient was managed by his local primary cardiologist with metoprolol and verapamil and referred to our institution at 12 years of age, asymptomatic in the setting of self-restricted physical activity due to ICD placement. His echocardiogram showed a severely hypertrophied ventricular septum, measuring 3.1 cm at end-diastole; dynamic LVOT obstruction with a peak resting gradient of 80 mmHg; and moderately restrictive left ventricular diastolic function. Cardiac magnetic resonance imaging showed patchy enhancement at the mid-septum. There was minimal ectopy on Holter monitoring, and an exercise stress test demonstrated a blunted heart rate and blood pressure response, though no ectopy was observed during exercise or recovery. Genetic testing subsequently revealed a pathogenic variant in the *MHY7* gene (c.1207 C>T, p.Arg403Trp).

**Figure 1:**
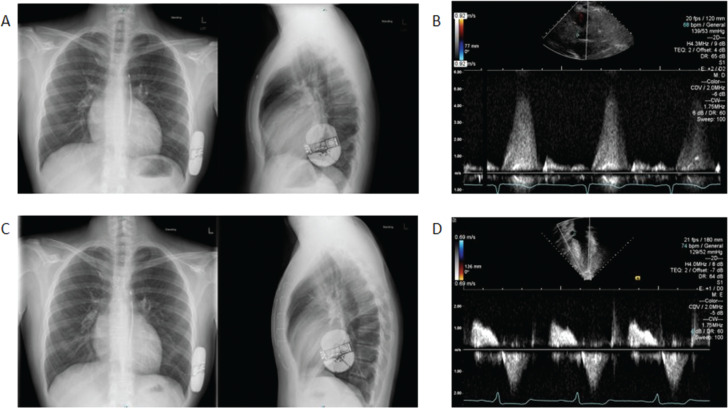
**A:** Anteroposterior and lateral chest X-rays just prior to sternotomy showing device placement with a PRAETORIAN score of 60 points, suggesting a low risk for conversion failure.^13^ **B:** High right parasternal long view showing an initial peak resting gradient of the left ventricular outflow tract of 80 mmHg. **C:** Anteroposterior and lateral chest X-rays post-myectomy showing stable positioning of the subcutaneous implantable cardioverter-defibrillator. **D:** Apical view at 2-month follow-up showing a minimal residual resting left ventricular outflow tract obstruction with a peak velocity of 2 m/s.

Based on the 2011 American College of Cardiology Foundation/American Heart Association guidelines, the patient met one major, one minor, and two modifying criteria for primary prevention with ICD placement.^12^ In the absence of a pacing need and following a discussion on the risks and benefits of transvenous ICDs and S-ICDs, the family elected for the S-ICD following S-ICD electrogram acceptability. An Emblem MRI S-ICD (Boston Scientific Corp., Marlborough, MA, USA) was implanted without complication using the two-incision technique. Defibrillation testing showed conversion to sinus rhythm with 65 J. The lead impedance was 72 Ω, with each vector being adequate. The device was programmed as conditional at 190 bpm and defibrillation at 220 bpm **(Figure 1)**.

The patient was monitored primarily by his primary cardiologist for a few years with adjustments to his metoprolol and verapamil. He had intermittent loss to follow-up with electrophysiology at our institution. Almost 4 years after device implantation, he had a syncopal event that was likely vasovagal in nature. There was no tachycardia or treatable arrhythmia detected by the device and no shock delivered during that episode.

**Figure 2:**
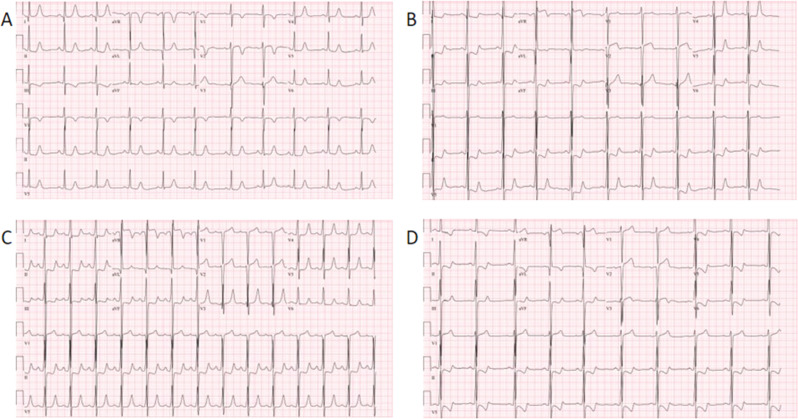
**A:** Initial electrocardiogram (ECG) on presentation with normal sinus rhythm. **B:** ECG when symptomatic with evidence of left ventricular hypertrophy (LVH) with QRS widening to 124 ms and T-wave inversion in lateral leads. **C:** Immediate postoperative ECG with evidence of LVH, a QRS of 118 ms, and repolarization abnormality. **D:** ECG 2 months postoperatively with LVH, a QRS of 128 ms, and repolarization abnormality.

At 17 years of age, he developed increasing chest pain and fatigue, including severe shortness of breath when walking up a flight of stairs. There were no episodes of tachycardia on his S-ICD, but an electrocardiogram (ECG) now demonstrated T-wave inversion in the lateral leads. His treatment at that time included 100 mg of metoprolol tartrate twice a day and 180 mg of verapamil extended release. It was recommended that the metoprolol tartrate be switched to long-acting metoprolol succinate. His echocardiogram showed a septal thickness, which remained stable at 3.1 cm at end-diastole; a peak resting gradient of his LVOT of 80 mmHg; and mild mitral regurgitation. With New York Heart Association class III symptoms and new ECG findings, he underwent cardiac catheterization, which demonstrated a long segmented myocardial bridge in his LAD and a 130-mmHg gradient in his LVOT without provocation **(Figure 2)**.

A multidisciplinary team including pediatric electrophysiology, pediatric heart failure, and congenital cardiothoracic surgery discussed the need for a surgical intervention on the patient’s septum and myocardial bridge of the LAD as well as the preservation of the S-ICD. Based on these discussions, it was decided that the proceeding was reasonable, despite the risk of a surgical left bundle branch block (LBBB), and that should postoperative sensing vectors prove problematic, system revision could be made at that time.

Prior to the incision, the cardiothoracic surgeon marked the location of the S-ICD lead underneath the skin. A median sternotomy was performed safely away from the lead, and care was taken to ensure that the sternal retractor did not impact the lead as well. Ultimately, the surgeons performed a myotomy/myectomy of the hypertrophied septum from underneath the right coronary cusp down to the anterior leaflet of the mitral valve. The surgeons then unroofed the LAD of almost 3 cm in length that was underneath 1-cm thickness of myocardium.

The postoperative transesophageal echocardiogram showed moderate septal hypertrophy with a marked reduction in the LVOT peak velocity to 1.6 m/s, predicting no significant residual gradient. There was no mitral regurgitation or aortic insufficiency. An S-ICD evaluation showed that all vectors were sensing appropriately prior to the patient leaving the operating room. An immediate postoperative ECG demonstrated normal sinus rhythm with voltage criteria for left ventricular hypertrophy and intraventricular conduction delay with a QRS duration of 118 ms. His postoperative course was uneventful, and he was continued on metoprolol and verapamil.

At a 2-month follow-up, the patient reported significant symptom improvement. An ECG revealed sinus bradycardia, left axis deviation, left ventricular hypertrophy, and QRS widening to 128 ms, though no change in morphology was observed. An echocardiogram demonstrated minimal residual resting LVOT obstruction with a peak velocity of 2 m/s. Interrogation of the device showed that all vectors were adequate, and the lead impedance was similar to implantation at 80 Ω.

The patient was again lost to follow-up with no remote transmissions. At 8 months postoperatively, he experienced a device discharge due to T-wave oversensing during sinus tachycardia. In telephone communication with the patient, it was found that he was now living on his own, and there have been some concerns about medication compliance and a switch back to metoprolol tartrate rather than long-acting metoprolol succinate. He has not returned to our office for evaluation and interrogation of his S-ICD.

## Discussion

This case demonstrates the feasibility of maintaining an S-ICD system function following a myectomy surgery in a pediatric HCM patient. It furthermore highlights the importance of a multidisciplinary discussion prior to surgical intervention.

The S-ICD remains an important option for young individuals without sinus or atrioventricular node dysfunction. Despite studies showing that the outcomes and overall complication rates of transvenous ICDs and S-ICDs are similar in children, recent literature suggests that there may be more lead-related complications, including difficult lead extractions, in younger patients with transvenous ICDs.^4–6,14,15^ It is possible that younger patients requiring an ICD may benefit from having an S-ICD initially placed for the preservation of blood vessels and to limit potential lead complications, including future extractions. Although previous studies found HCM to be a risk factor for failing the screening for S-ICD placement, our patient successfully had S-ICD electrogram acceptability.^16^ Given that our patient received the S-ICD as a younger adolescent, we considered that there could be further hypertrophy of his ventricular septum, potentially leading to ECG changes or requirements for interventions such as a septal myectomy.^17,18^ As always, using a shared decision-making approach with the family, reviewing these and other salient discussion points is critical.

Regarding the initial placement of the S-ICD electrode, we followed the traditional placement, approximately 1–2 cm left of the midline though—not so lateral as to leave the sternum proper. This provided for adequate distance away from the mid-sternotomy incision with the added benefit of protection by the sternal retractors throughout the operation. Care to maintain a midline approach for the sternotomy is important for optimal postoperative healing. The lead was placed left of the sternum, as per our suggested technique, allowing the cardiothoracic surgeon the ability to preserve the lead during the future median sternotomy, and the postoperative PRAETORIAN score was also suggestive of a low risk for conversion failure.^14^

Once the patient became symptomatic and had T-wave inversion on ECG, he underwent a cardiac catheterization, which elucidated the myocardial bridging of his LAD and significant LVOT obstruction. This catheterization, along with a chest X-ray and an echocardiogram, provided the data for a multidisciplinary team discussion. Discussion topics included alerting the surgeon of the lead placement regarding the sternum and change in S-ICD vector fidelity due to conduction abnormalities following myectomy. Previous case reports in adult literature have shown that sternotomies could be accomplished without damaging the subcutaneous lead.^7,8^ Cui et al. demonstrated that, in the adult population with HCM undergoing a myectomy, LBBB developed in almost 39% of cases.^18^ A case report presented a pediatric patient with an S-ICD who developed LBBB intraoperatively after a myomectomy, and subsequently, the patient’s device was removed due to changes in the electrogram morphology.^9^ Our patient did not develop a significant surgical conduction abnormality, and his S-ICD showed adequate vectors prior to leaving the operating room and at 2 months of follow-up. Though his QRS had widened slightly from 118 to 128 ms at 2 months, it had no change in morphology, and prior ECGs showed widths of 122 and 124 ms, suggesting measurement variance. Nevertheless, LVOT myectomy remains a risk for surgical LBBB, which could complicate S-ICD sensing and must be considered in surgical planning.

**Figure 3:**
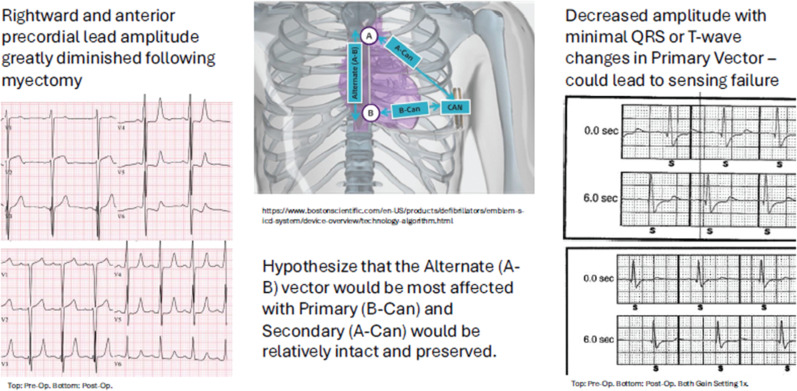
Subcutaneous implantable cardioverter-defibrillator sensing changes in pediatric hypertrophic cardiomyopathy following myectomy.

At later follow-up, our patient experienced an inappropriate shock 8 months after myectomy due to T-wave oversensing during sinus tachycardia. One multicenter study of adult patients with HCM had 5 out of 88 patients receiving an inappropriate shock most often because of sinus tachycardia and/or T-wave oversensing.^19^ Studies in adult and pediatric patients with HCM found similar rates of inappropriate shocks between transvenous ICDs and S-ICDs.^4–6,14^ Furthermore, there are new algorithms in S-ICDs that have resulted in decreased inappropriate shocks.^19^ Oversensing and undersensing remain a concern in the pediatric population due to growth over time in addition to the important changes that occur following a myectomy surgery **(Figure 3)**. Close follow-up with the family, primary care provider, and additional subspecialty providers in addition to improved methods for medication adherence must be emphasized. Despite mixed evidence for using an exercise stress test to monitor T-wave oversensing, this strategy may be used to improve device sensing.^20^ Importantly, we recognize that this single case report may not be generalizable across global centers, though we believe that insights to assist in a successful surgery are present. With the growing population of adolescent patients with HCM receiving S-ICD placement, additional study of this population regarding outcomes post-sternotomy is indicated.

## Conclusion

Surgical myectomy following primary prevention S-ICD placement in pediatric HCM patients can be performed with excellent hemodynamic outcomes and no significant effects on S-ICD performance in the absence of LBBB. Close follow-up is required to monitor for any ECG changes that may lead to inappropriate shocks.
